# Supplementary material for: Regenerative base editing enables deep lineage recording
Source: bioRxiv. 2026 Feb 7:2026.02.06.703857. Preprint. [Version 1] doi: 10.64898/2026.02.06.703857 (PMC12889630; doi:10.64898/2026.02.06.703857)
Supplement: 1 [file NIHPP2026.02.06.703857V1-supplement-1.pdf]

bioRxiv preprint doi: https://doi.org/10.64898/2026.02.06.703857; this version posted February 7, 2026. The copyright holder for this preprint (which was not certified by peer review) is the author/funder, who has granted bioRxiv a license to display the preprint in perpetuity. It is made available under aCC-BY 4.0 International license.

Supplemental Figures

bioRxiv preprint doi: https://doi.org/10.64898/2026.02.06.703857; this version posted February 7, 2026. The copyright holder for this preprint (which was not certified by peer review) is the author/funder, who has granted bioRxiv a license to display the preprint in perpetuity. It is made available under aCC-BY 4.0 International license.

Supplemental Figure 1: The hypercascade typically outperforms Cas9 based systems of equal length in simulations.

Cas9 can produce multiple editing outcomes where base editors produce only a single A-to-G transition. We used a simplified model of Cas9 editing by allowing for each target state to take one of 8 different editing outcomes (see methods). Holding tree depth constant, the hypercascade system outperforms a simulated independent Cas9 target array with comparable sequence length over a variety of edit rates and copy numbers, but underperforms slightly for low edit rates and tree depths.

bioRxiv preprint doi: https://doi.org/10.64898/2026.02.06.703857; this version posted February 7, 2026. The copyright holder for this preprint (which was not certified by peer review) is the author/funder, who has granted bioRxiv a license to display the preprint in perpetuity. It is made available under aCC-BY 4.0 International license.

A Hypercascade arrays typically outperform Cas9 arrays of comparable length

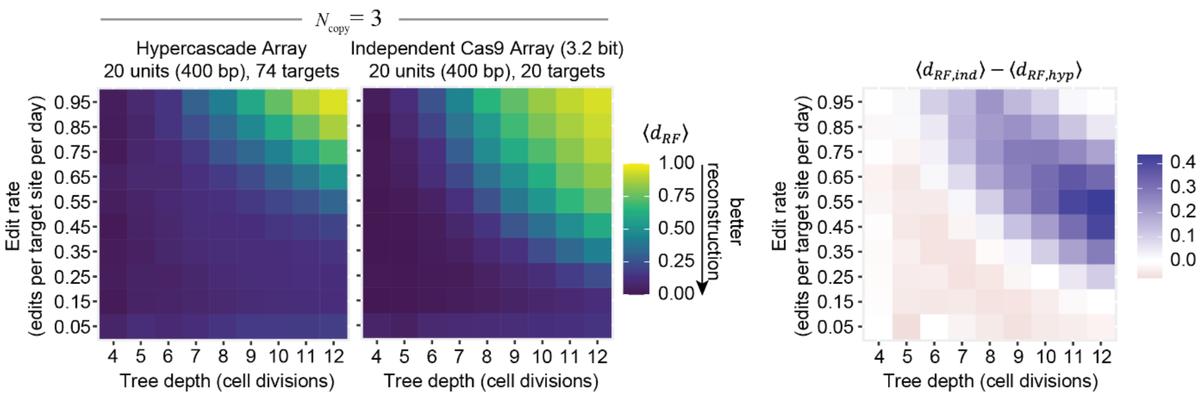

bioRxiv preprint doi: https://doi.org/10.64898/2026.02.06.703857; this version posted February 7, 2026. The copyright holder for this preprint (which was not certified by peer review) is the author/funder, who has granted bioRxiv a license to display the preprint in perpetuity. It is made available under aCC-BY 4.0 International license.

Supplemental Figure 2: Mismatch edit rates can be estimated.
(A) Bulk sequencing data can be used to estimate edit rates through either protospacer or PAM site mismatches under several assumptions. **(B)** This is possible by making the assumption that any edit pattern was generated in the most likely ordering of events.

bioRxiv preprint doi: https://doi.org/10.64898/2026.02.06.703857; this version posted February 7, 2026. The copyright holder for this preprint (which was not certified by peer review) is the author/funder, who has granted bioRxiv a license to display the preprint in perpetuity. It is made available under aCC-BY 4.0 International license.

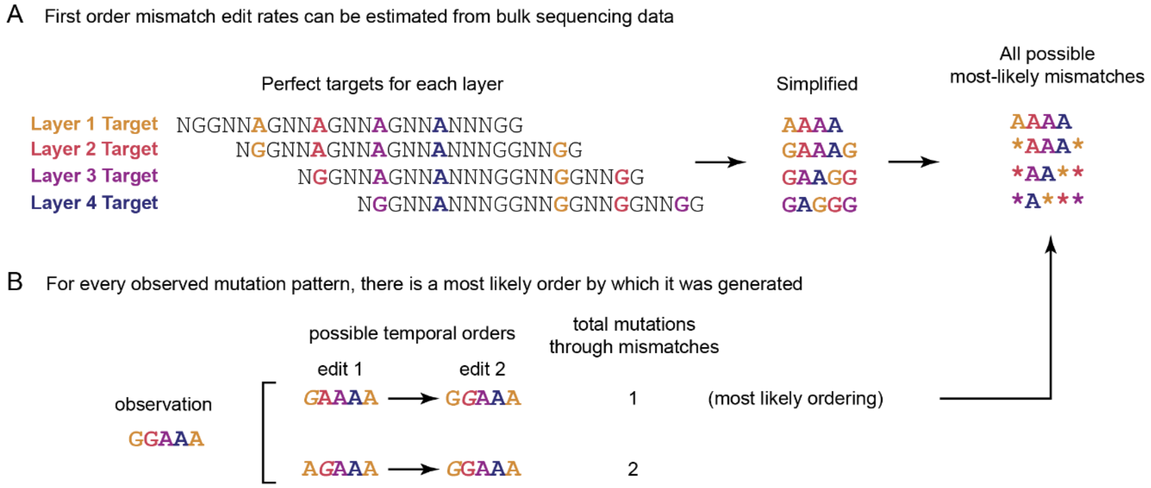

bioRxiv preprint doi: https://doi.org/10.64898/2026.02.06.703857; this version posted February 7, 2026. The copyright holder for this preprint (which was not certified by peer review) is the author/funder, who has granted bioRxiv a license to display the preprint in perpetuity. It is made available under aCC-BY 4.0 International license.

Supplemental Figure 3: The editing process generates potential mismatch targets dynamically. To estimate mutation rates for a given sequence context, we need to know the number of target sites seen by the editor over time. This can be extracted from bulk sequencing data, both in Patski cells for designs 1 **(A)**, 2 **(B)**, and 3 **(C)**, as well as for design 1 in hiPSCs **(D)**.

bioRxiv preprint doi: https://doi.org/10.64898/2026.02.06.703857; this version posted February 7, 2026. The copyright holder for this preprint (which was not certified by peer review) is the author/funder, who has granted bioRxiv a license to display the preprint in perpetuity. It is made available under aCC-BY 4.0 International license.

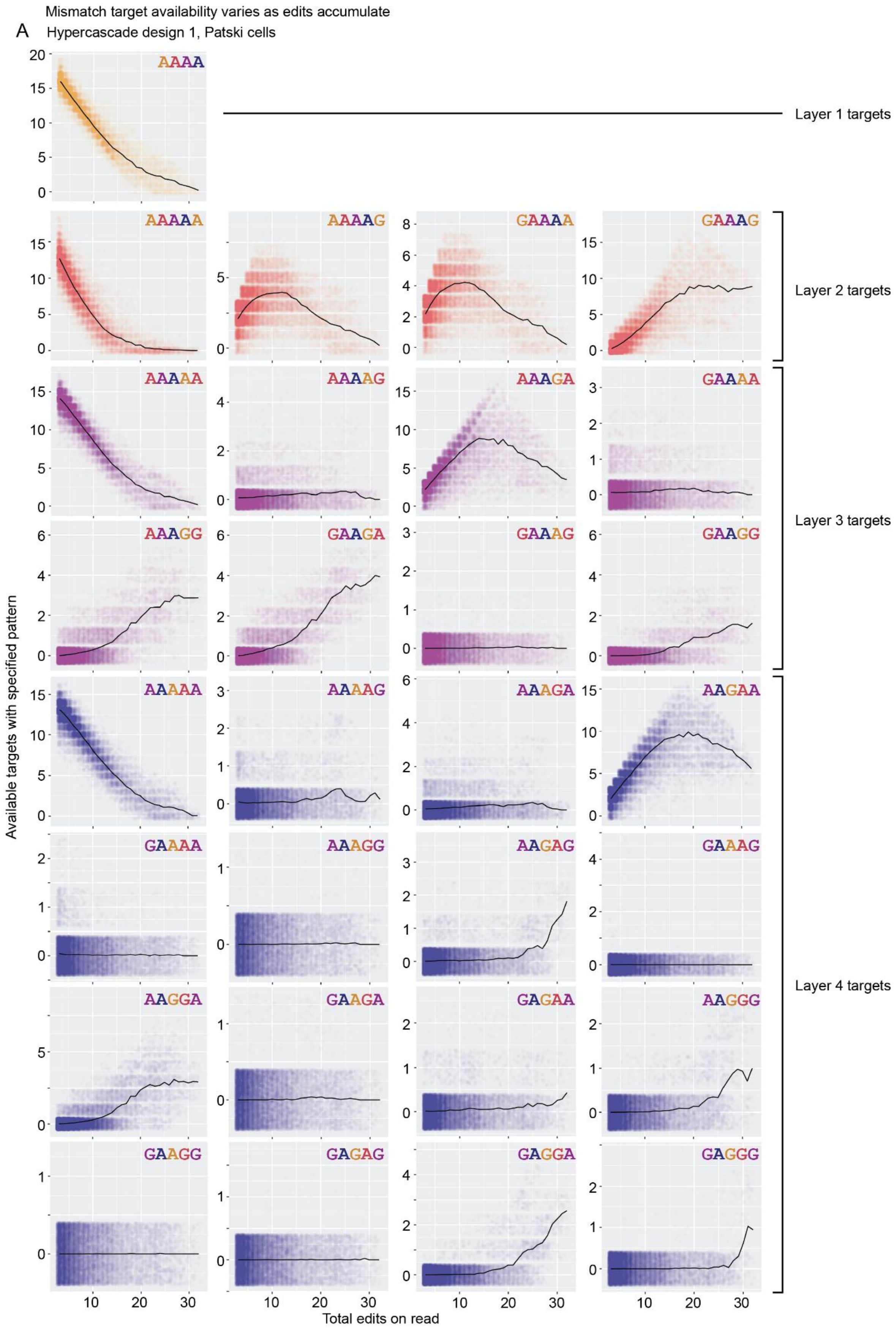

bioRxiv preprint doi: https://doi.org/10.64898/2026.02.06.703857; this version posted February 7, 2026. The copyright holder for this preprint (which was not certified by peer review) is the author/funder, who has granted bioRxiv a license to display the preprint in perpetuity. It is made available under aCC-BY 4.0 International license.

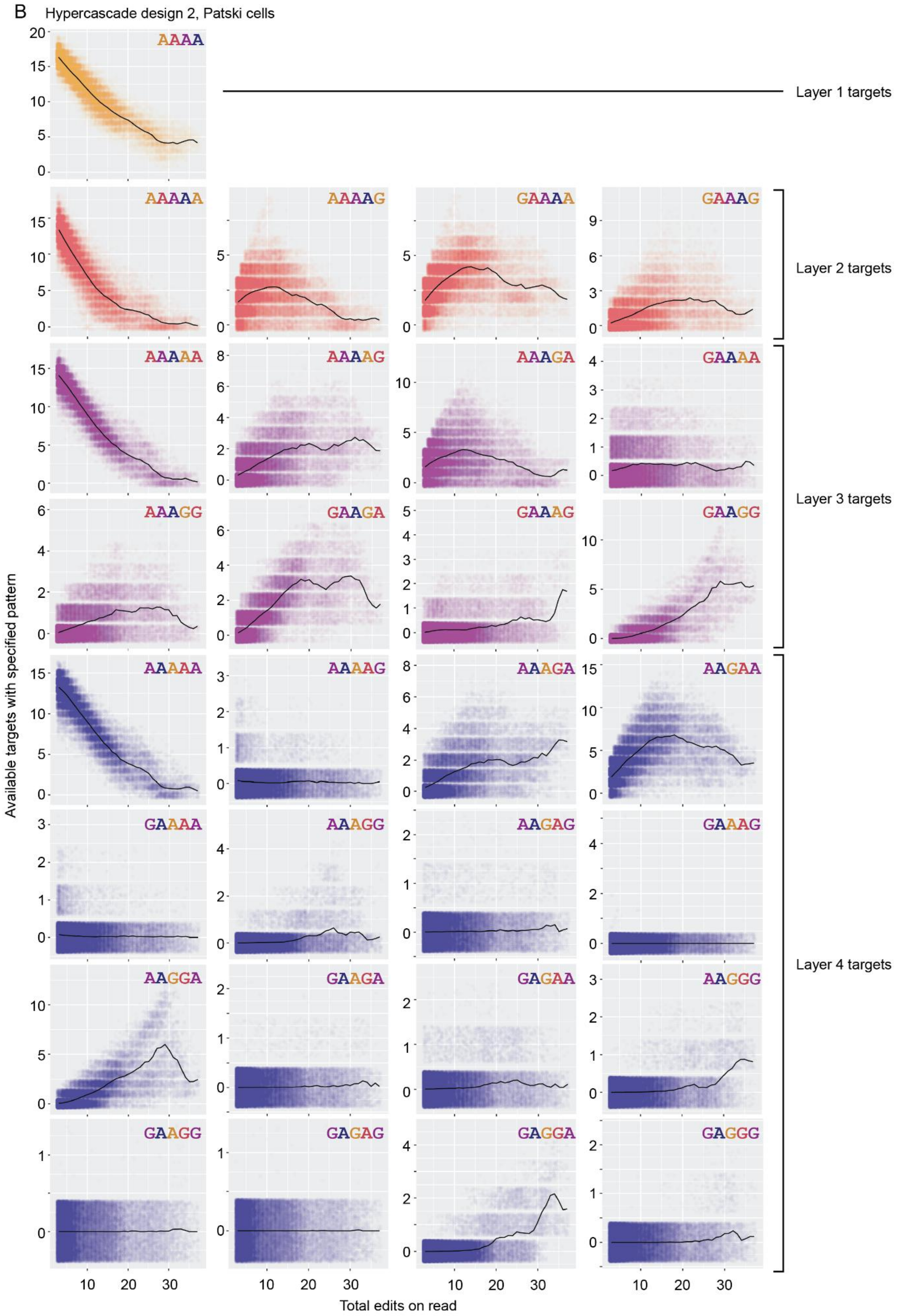

bioRxiv preprint doi: https://doi.org/10.64898/2026.02.06.703857; this version posted February 7, 2026. The copyright holder for this preprint (which was not certified by peer review) is the author/funder, who has granted bioRxiv a license to display the preprint in perpetuity. It is made available under aCC-BY 4.0 International license.

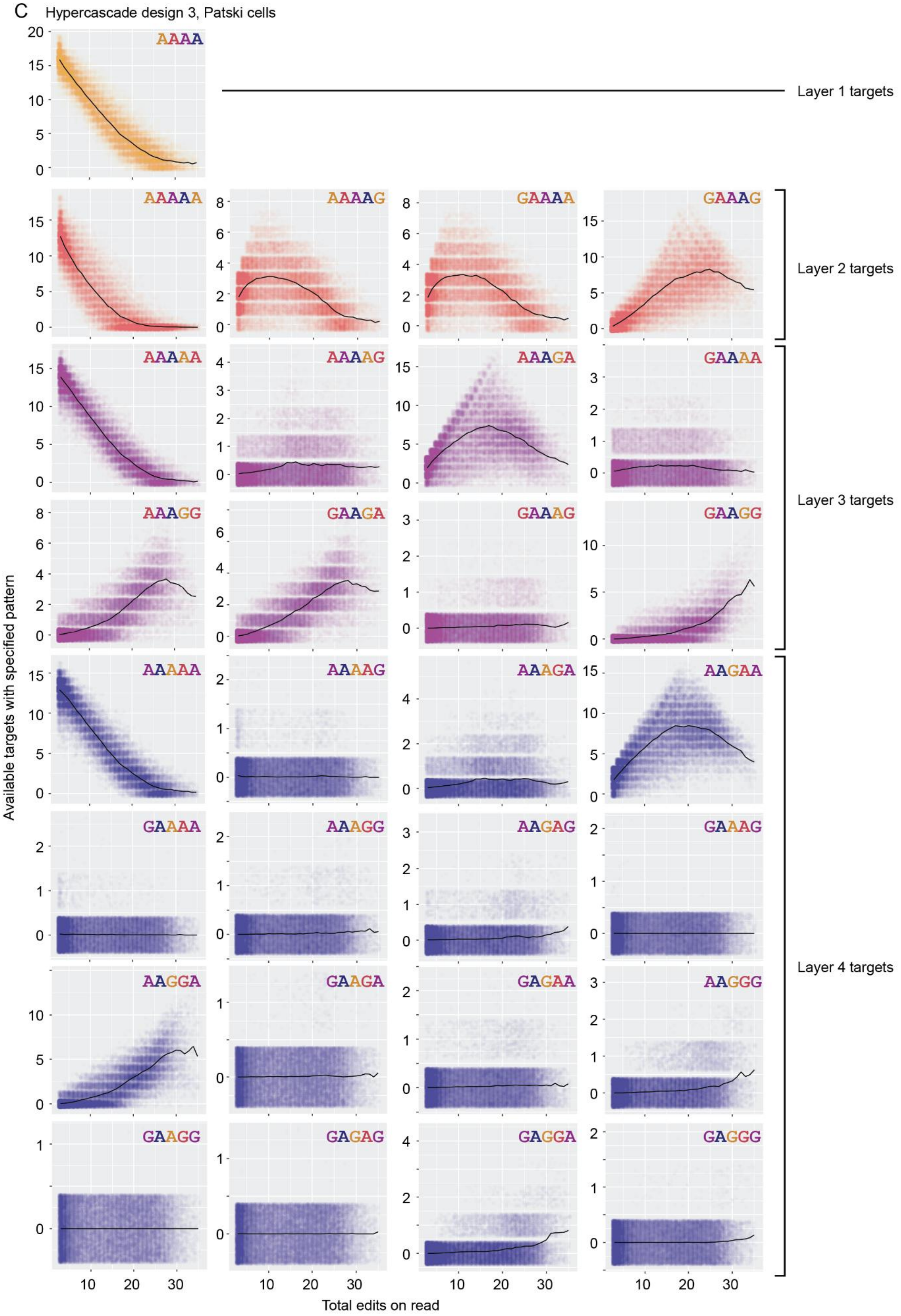

bioRxiv preprint doi: https://doi.org/10.64898/2026.02.06.703857; this version posted February 7, 2026. The copyright holder for this preprint (which was not certified by peer review) is the author/funder, who has granted bioRxiv a license to display the preprint in perpetuity. It is made available under aCC-BY 4.0 International license.

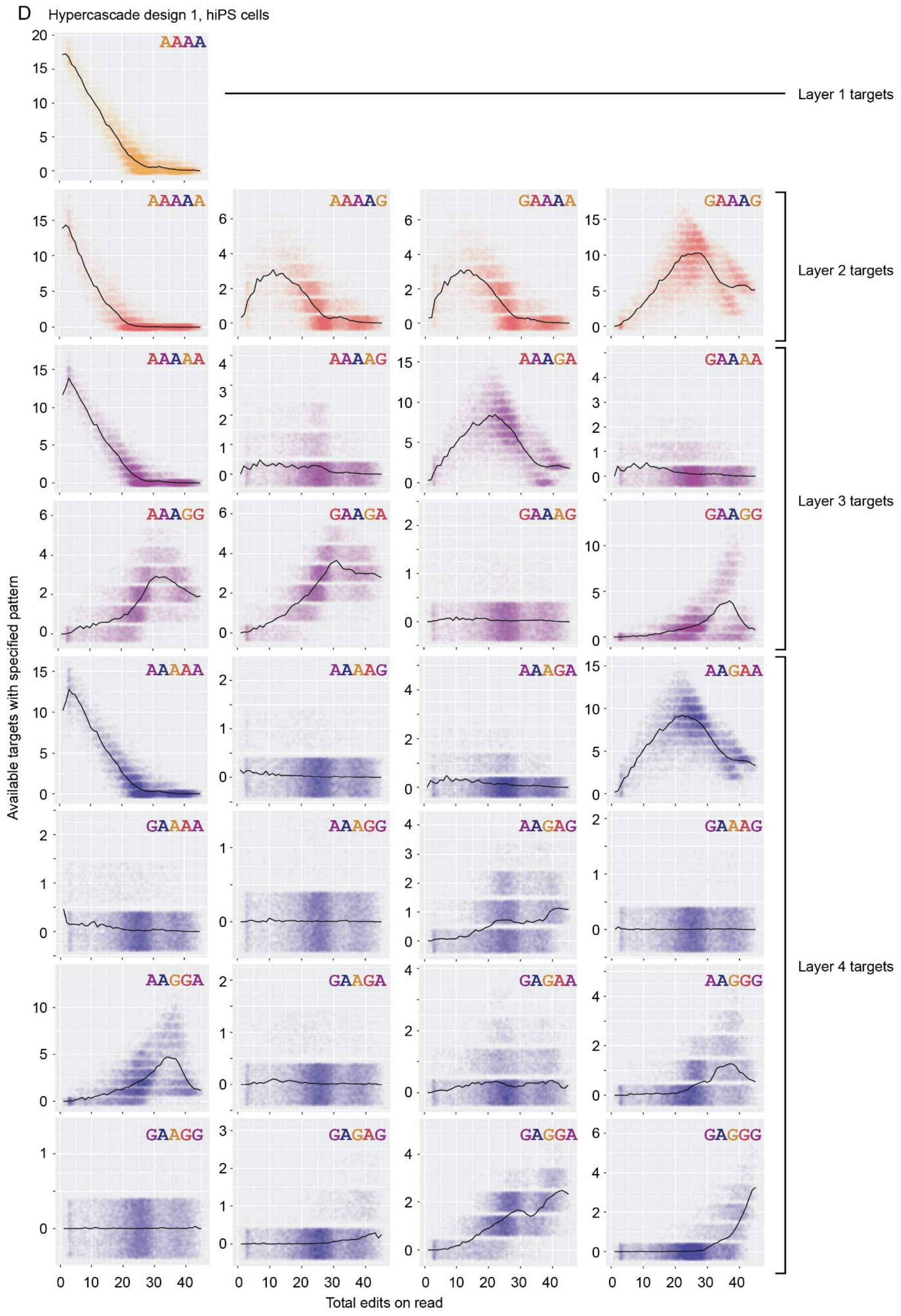

bioRxiv preprint doi: https://doi.org/10.64898/2026.02.06.703857; this version posted February 7, 2026. The copyright holder for this preprint (which was not certified by peer review) is the author/funder, who has granted bioRxiv a license to display the preprint in perpetuity. It is made available under aCC-BY 4.0 International license.

Supplemental Figure 4: Mismatches decrease the rate of editing across all layers. Mismatch edit rates for many sequence contexts can be estimated from bulk sequencing data. Typically gRNA-protospacer mismatches are estimated to decrease edit rate, with multiple mismatches and mismatches proximal to the PAM site having greater effects. Error bars represent standard error.

bioRxiv preprint doi: https://doi.org/10.64898/2026.02.06.703857; this version posted February 7, 2026. The copyright holder for this preprint (which was not certified by peer review) is the author/funder, who has granted bioRxiv a license to display the preprint in perpetuity. It is made available under aCC-BY 4.0 International license.

bioRxiv preprint doi: https://doi.org/10.64898/2026.02.06.703857; this version posted February 7, 2026. The copyright holder for this preprint (which was not certified by peer review) is the author/funder, who has granted bioRxiv a license to display the preprint in perpetuity. It is made available under aCC-BY 4.0 International license.

A Editing rate is reduced to varying extent bymismatches between the gRNA and target site

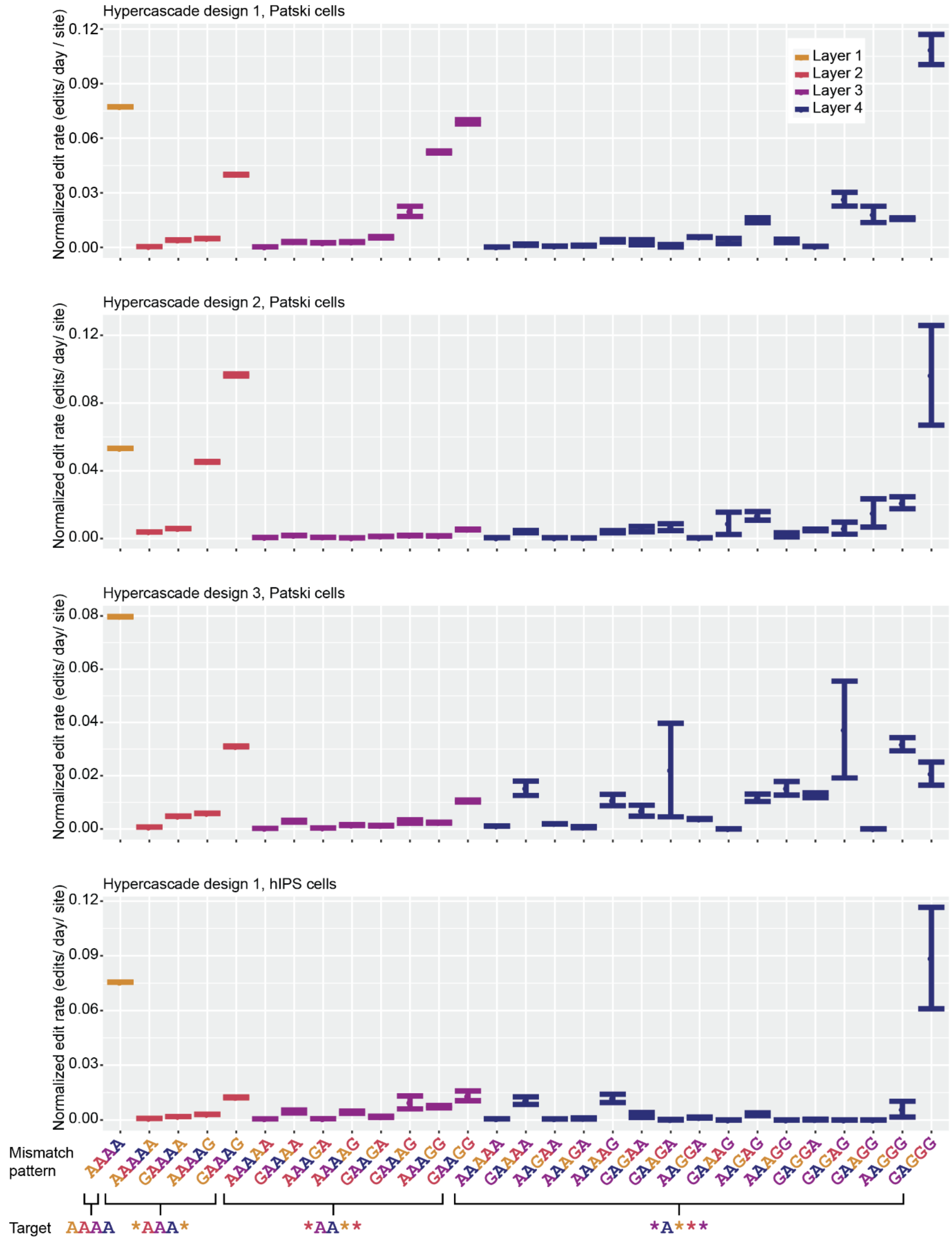

bioRxiv preprint doi: https://doi.org/10.64898/2026.02.06.703857; this version posted February 7, 2026. The copyright holder for this preprint (which was not certified by peer review) is the author/funder, who has granted bioRxiv a license to display the preprint in perpetuity. It is made available under aCC-BY 4.0 International license.

Supplemental Figure 5: Bayesian analysis and bootstrapping reveal confidently reconstructed phylogenetic relationships among thousands of hiPSCs. (A) 90 randomly sampled hypercascades were analyzed using BEAST2 to assess uncertainty in the resulting lineage reconstruction. We identify high transfer scores for a number of clades across the randomly chosen barcodes. **(B)** An alternative method, phylogenetic bootstrapping with UPGMA phylogenetic reconstruction, reveals multiple confidently assigned clades across the full tree. In part **(B)**, only clades with at least 30% bootstrap transfer score are visualized for clarity.

bioRxiv preprint doi: https://doi.org/10.64898/2026.02.06.703857; this version posted February 7, 2026. The copyright holder for this preprint (which was not certified by peer review) is the author/funder, who has granted bioRxiv a license to display the preprint in perpetuity. It is made available under aCC-BY 4.0 International license.

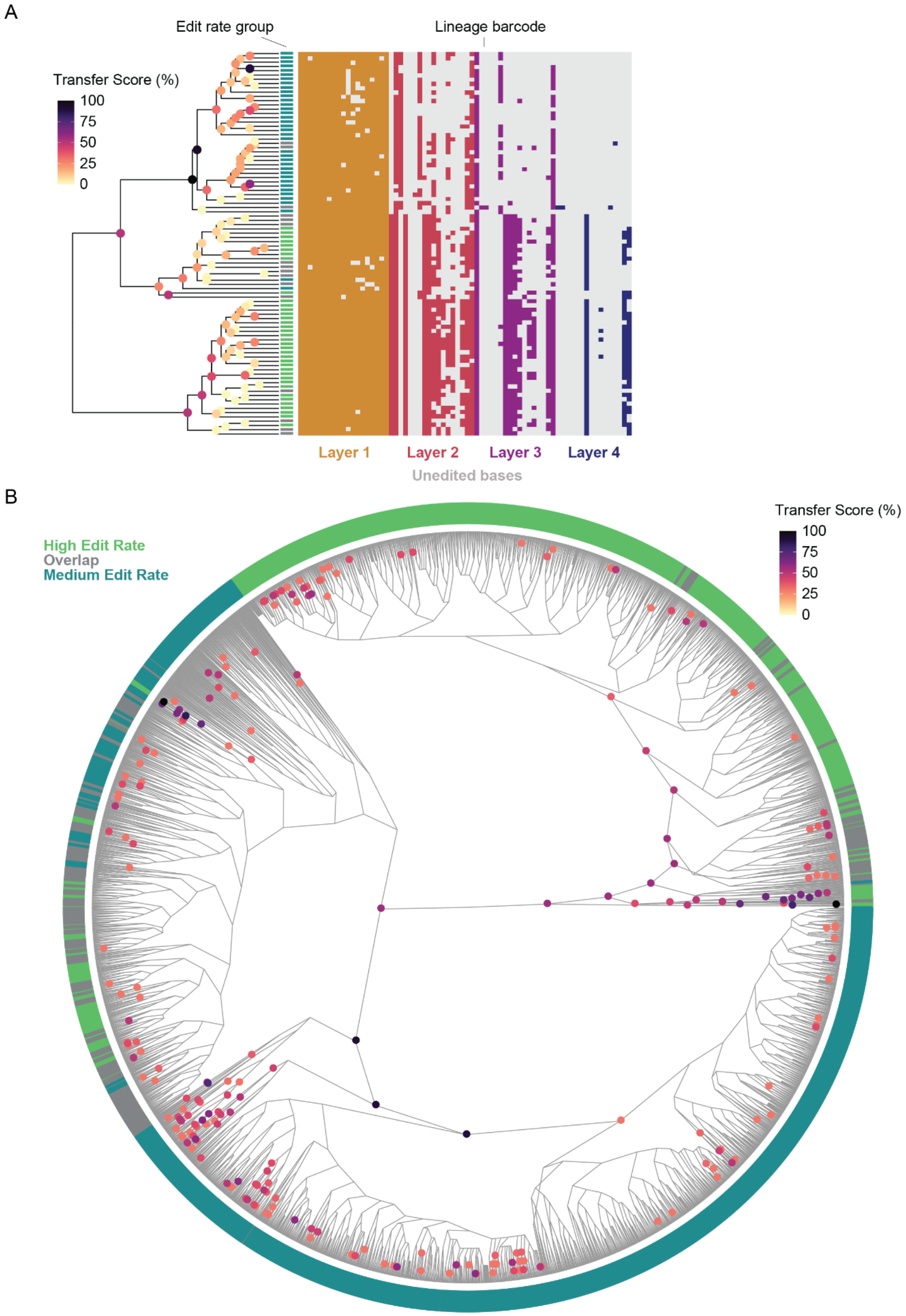

bioRxiv preprint doi: https://doi.org/10.64898/2026.02.06.703857; this version posted February 7, 2026. The copyright holder for this preprint (which was not certified by peer review) is the author/funder, who has granted bioRxiv a license to display the preprint in perpetuity. It is made available under aCC-BY 4.0 International license.

Supplemental Figure 6: Editing over long times can lead to target array collapse. Hypercascade sequences are amplified from bulk genomic DNA and expected to produce a 500 bp product.

bioRxiv preprint doi: https://doi.org/10.64898/2026.02.06.703857; this version posted February 7, 2026. The copyright holder for this preprint (which was not certified by peer review) is the author/funder, who has granted bioRxiv a license to display the preprint in perpetuity. It is made available under aCC-BY 4.0 International license.

Hypercascade barcodes can collapse with extended editing durations

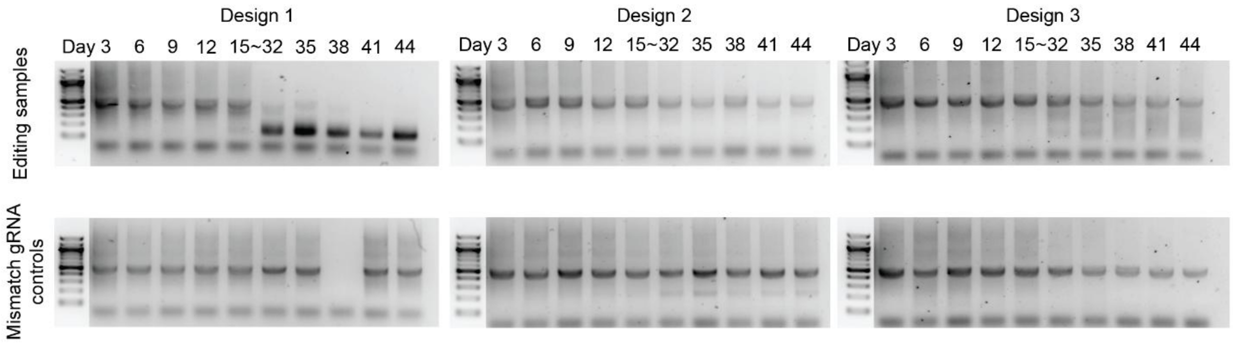

bioRxiv preprint doi: https://doi.org/10.64898/2026.02.06.703857; this version posted February 7, 2026. The copyright holder for this preprint (which was not certified by peer review) is the author/funder, who has granted bioRxiv a license to display the preprint in perpetuity. It is made available under aCC-BY 4.0 International license.

Supplemental Figure 7: Open chromatin edits more rapidly than closed chromatin.
(A) 12 targets dispersed across the X chromosome were targeted for base editing over a 1-5 day time course, transfecting either 15 or 30 nM of gRNA into cells constitutively expressing ABE. Targets were selected to have differential chromatin accessibility on the active and inactive X alleles based on existing ATAC sequencing data. Editing was quantified for each allele to determine whether chromatin context impacts ABE edit rate. **(B)** An additional 10 targets were selected and targeted for editing within and around a single differential ATAC peak from panel A. Cells were transfected with 30 nM gRNA only in this case.

bioRxiv preprint doi: https://doi.org/10.64898/2026.02.06.703857; this version posted February 7, 2026. The copyright holder for this preprint (which was not certified by peer review) is the author/funder, who has granted bioRxiv a license to display the preprint in perpetuity. It is made available under aCC-BY 4.0 International license.

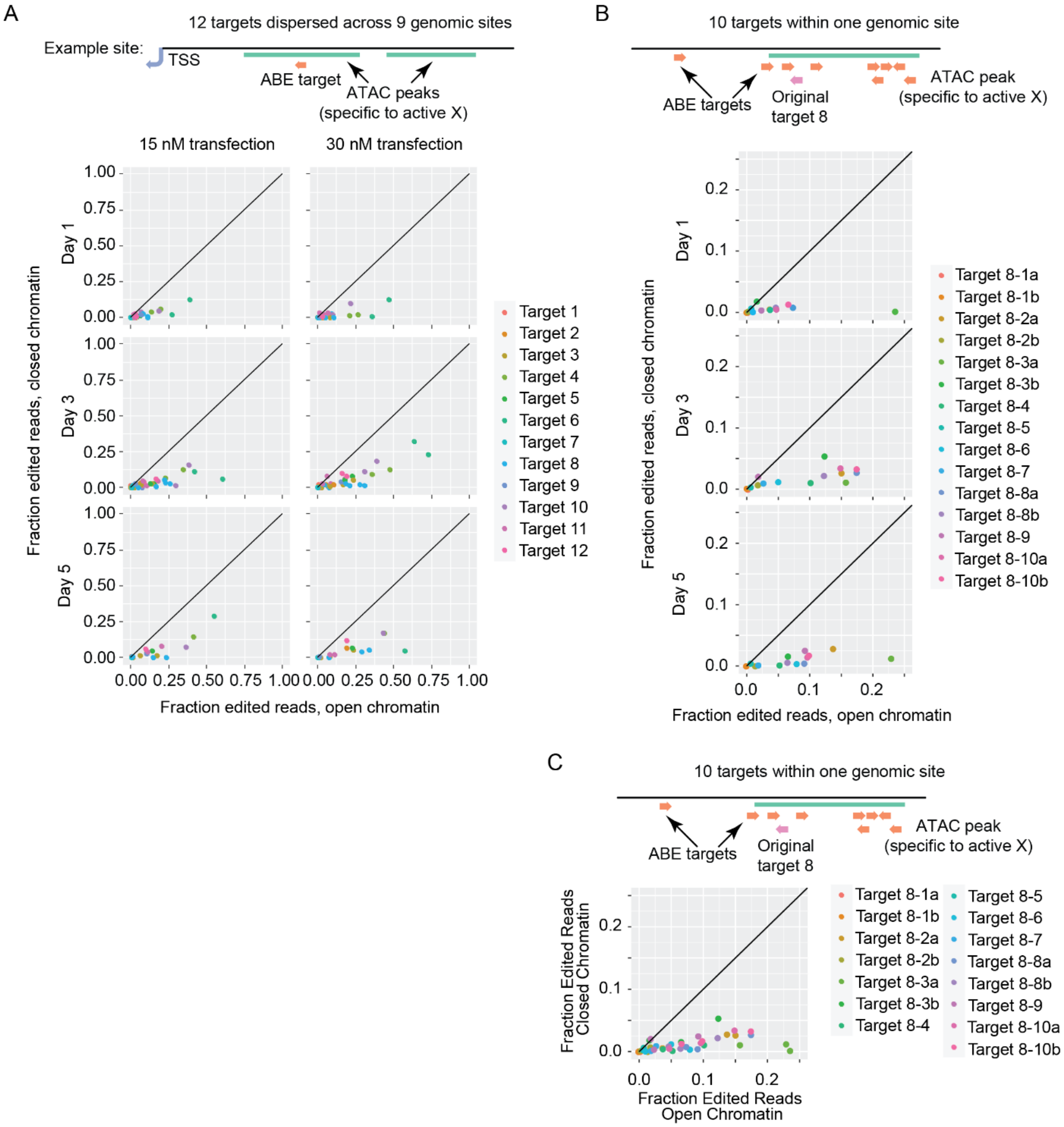

bioRxiv preprint doi: https://doi.org/10.64898/2026.02.06.703857; this version posted February 7, 2026. The copyright holder for this preprint (which was not certified by peer review) is the author/funder, who has granted bioRxiv a license to display the preprint in perpetuity. It is made available under aCC-BY 4.0 International license.

Supplemental Table 1: Hypercascade target sequences investigated in this study.

	20mer Repeat	Layer 1 gRNA	Layer 2 gRNA	Layer 3 gRNA	Layer 4 gRNA
Target 1	AGGACAGTCAGACAGTCATG…	AGGACAGTCAGACAGTCATG	CGGTCAGACAGTCATGAGGA	CGGACAGTCATGAGGACGGT	CGGTCATGAGGACGGTCGGA
Target 2	AGGTCAGACAGTCAGACACA…	AGGTCAGACAGTCAGACACA	CGGACAGTCAGACACAAGGT	CGGTCAGACACAAGGTCGGA	CGGACACAAGGTCGGACGGT
Target 3	AGGTCAGTCAGTAAGTAACG…	AGGTCAGTCAGTAAGTAACG	CGGTCAGTAAGTAACGAGGT	CGGTAAGTAACGAGGTCGGT	AGGTAACGAGGTCGGTCGGT

bioRxiv preprint doi: https://doi.org/10.64898/2026.02.06.703857; this version posted February 7, 2026. The copyright holder for this preprint (which was not certified by peer review) is the author/funder, who has granted bioRxiv a license to display the preprint in perpetuity. It is made available under aCC-BY 4.0 International license.

Supplemental Table 2: Primer sequences used in this study.

bioRxiv preprint doi: https://doi.org/10.64898/2026.02.06.703857; this version posted February 7, 2026. The copyright holder for this preprint (which was not certified by peer review) is the author/funder, who has granted bioRxiv a license to display the preprint in perpetuity. It is made available under aCC-BY 4.0 International license.

**Amplicon**	**Primer Sequence**
g01 (Supplemental Figure 6A), Forward, with Illumina Adapters	TCGTCGGCAGCGTCAGATGTGTATAAGAGACAGGGTAACCAAAGGCTGGAGCTC
g01 (Supplemental Figure 6A), Reverse, with Illumina Adapters	GTCTCGTGGGCTCGGAGATGTGTATAAGAGACAGCCAGCGCCCACTATTAAAGTACC
g02 (Supplemental Figure 6A), Forward, with Illumina Adapters	TCGTCGGCAGCGTCAGATGTGTATAAGAGACAGTGTGATGTGCATCCAGTAATGTTCC
g02 (Supplemental Figure 6A), Reverse, with Illumina Adapters	GTCTCGTGGGCTCGGAGATGTGTATAAGAGACAGGAGTTGTCCAAGGCCACTGAG
g03 (Supplemental Figure 6A), Forward, with Illumina Adapters	TCGTCGGCAGCGTCAGATGTGTATAAGAGACAGCGTTTCAGCCAAAGTACTCCTCAC
g03 (Supplemental Figure 6A), Reverse, with Illumina Adapters	GTCTCGTGGGCTCGGAGATGTGTATAAGAGACAGGGCTAGCTCCTGACTGATCAG
g04 (Supplemental Figure 6A), Forward, with Illumina Adapters	TCGTCGGCAGCGTCAGATGTGTATAAGAGACAGGGTACGTGCCCCAAGATAACC
g04 (Supplemental Figure 6A), Reverse, with Illumina Adapters	GTCTCGTGGGCTCGGAGATGTGTATAAGAGACAGCTTGCCCTGCTCTAGCCTTG
g05 (Supplemental Figure 6A), Forward, with Illumina Adapters	TCGTCGGCAGCGTCAGATGTGTATAAGAGACAGCTGGCTAATGCCAAGGACTCTG
g05 (Supplemental Figure 6A), Reverse, with Illumina Adapters	GTCTCGTGGGCTCGGAGATGTGTATAAGAGACAGGGCTGGCCAAAGGACCATC

bioRxiv preprint doi: https://doi.org/10.64898/2026.02.06.703857; this version posted February 7, 2026. The copyright holder for this preprint (which was not certified by peer review) is the author/funder, who has granted bioRxiv a license to display the preprint in perpetuity. It is made available under aCC-BY 4.0 International license.

g06 (Supplemental Figure 6A), Forward, with Illumina Adapters	TCGTCGGCAGCGTCAGATGTGTATAAGAGACAGCTCAAAGAAGGCCAAAGCCAAG
g06 (Supplemental Figure 6A), Reverse, with Illumina Adapters	GTCTCGTGGGCTCGGAGATGTGTATAAGAGACAGCTCATCCGGATCCTCACCAATC
g07 (Supplemental Figure 6A), Forward, with Illumina Adapters	TCGTCGGCAGCGTCAGATGTGTATAAGAGACAGGATAAACCTAGACAATGCTTCCAGG
g07 (Supplemental Figure 6A), Reverse, with Illumina Adapters	GTCTCGTGGGCTCGGAGATGTGTATAAGAGACAGCCACTGAGCTACATTCTGAGTCC
g08 (Supplemental Figure 6A), Forward, with Illumina Adapters	TCGTCGGCAGCGTCAGATGTGTATAAGAGACAGCGAGCAAAACTAGGAGTCTTTCC
g08 (Supplemental Figure 6A), Reverse, with Illumina Adapters	GTCTCGTGGGCTCGGAGATGTGTATAAGAGACAGGCTCTGGCTTCTGTAGTGGG
g09 (Supplemental Figure 6A), Forward, with Illumina Adapters	TCGTCGGCAGCGTCAGATGTGTATAAGAGACAGCTCTGTTTATGCACAGTGCCG
g09 (Supplemental Figure 6A), Reverse, with Illumina Adapters	GTCTCGTGGGCTCGGAGATGTGTATAAGAGACAGCTTGACGACACCCACTGGC
g10 (Supplemental Figure 6A), Forward, with Illumina Adapters	TCGTCGGCAGCGTCAGATGTGTATAAGAGACAGCAGGCAAGAAACTTCTGCCAC
g10 (Supplemental Figure 6A), Reverse, with Illumina Adapters	GTCTCGTGGGCTCGGAGATGTGTATAAGAGACAGGCTGTGATTGGTATATGAGGCAGG

bioRxiv preprint doi: https://doi.org/10.64898/2026.02.06.703857; this version posted February 7, 2026. The copyright holder for this preprint (which was not certified by peer review) is the author/funder, who has granted bioRxiv a license to display the preprint in perpetuity. It is made available under aCC-BY 4.0 International license.

g11 (Supplemental Figure 6A), Forward, with Illumina Adapters	TCGTCGGCAGCGTCAGATGTGTATAAGAGACAGCTGCATTGCATACCCAGAGTTTC
g11 (Supplemental Figure 6A), Reverse, with Illumina Adapters	GTCTCGTGGGCTCGGAGATGTGTATAAGAGACAGGCTCAGGGTAACACACATGAGC
g12 (Supplemental Figure 6A), Forward, with Illumina Adapters	TCGTCGGCAGCGTCAGATGTGTATAAGAGACAGGCTCCGAGGTACTGGAAAGG
g12 (Supplemental Figure 6A), Reverse, with Illumina Adapters	GTCTCGTGGGCTCGGAGATGTGTATAAGAGACAGGAAGAGTCAAAGCTGGGACACAG
Long Amplicon for Region 8 with UMI (Supplemental Figure 6B), Linear PCR Primer, with Illumina Adapters	CAGCGTCAGATGTGTATAAGAGACAGNNNNNNNNNNGCCACATGATGGCTCACAAC
Long Amplicon for Region 8 with UMI (Supplemental Figure 6B), Exponential PCR Forward Primer, with Illumina Adapters	TCGTCGGCAGCGTCAGATGTGTATAAGAGACAG
Hypercascade target, Forward, with Illumina Adapters	TCGTCGGCAGCGTCAGATGTGTATAAGAGACAGCAGTGCAGTGCTTGATAACAGG
Hypercascade target, Reverse, with Illumina Adapters	GTCTCGTGGGCTCGGAGATGTGTATAAGAGACAGGATAGTCTGCGTAAAATTGACGCATG

bioRxiv preprint doi: https://doi.org/10.64898/2026.02.06.703857; this version posted February 7, 2026. The copyright holder for this preprint (which was not certified by peer review) is the author/funder, who has granted bioRxiv a license to display the preprint in perpetuity. It is made available under aCC-BY 4.0 International license.

Supplemental Table 3: Homology sequences for Patski guide RNA targets (Figure 5D and Supplemental Figure 6).

bioRxiv preprint doi: https://doi.org/10.64898/2026.02.06.703857; this version posted February 7, 2026. The copyright holder for this preprint (which was not certified by peer review) is the author/funder, who has granted bioRxiv a license to display the preprint in perpetuity. It is made available under aCC-BY 4.0 International license.

gRNA Target	Homology Sequence
g01 (Supplemental Figure 6A)	CGTCGACTGCTGGTCACGTG
g02 (Supplemental Figure 6A)	TGTCCATCTCTGGACACAGG
g03 (Supplemental Figure 6A)	TCCAAAATGAGTAGAGACCG
g04 (Supplemental Figure 6A)	TAGGACTGTGTTACAAACAG
g05 (Supplemental Figure 6A)	TACCCAGCTCTTTGTCACAG
g06 (Supplemental Figure 6A)	ACACACCAAGAGATAGAGAG
g07 (Supplemental Figure 6A)	AAGTCAATTAAATGGCTGCA
g08 (Supplemental Figure 6A)	CGGAACACTGACCATGGTCA
g09 (Supplemental Figure 6A)	GCCGACTTTGAAATTCGAGG
g10 (Supplemental Figure 6A)	ACCCCAGTGAGCAATGACAG
g11 (Supplemental Figure 6A)	TTGGAGAAAGCACGTCACTG
g12 (Supplemental Figure 6A)	ACTGTACCGAGCTAGCTCGG
gRNA 1 (Supplemental Figure 6B)	TAGTAAACATTATGCACATA
gRNA 2 (Supplemental Figure 6B)	CATCAAACAACCTTAATAAG
gRNA 3 (Supplemental Figure 6B)	TTTGAAGTGAGCCCTGACCA
gRNA 4 (Supplemental Figure 6B)	Same as g08 (Supplemental Figure 6A)

bioRxiv preprint doi: https://doi.org/10.64898/2026.02.06.703857; this version posted February 7, 2026. The copyright holder for this preprint (which was not certified by peer review) is the author/funder, who has granted bioRxiv a license to display the preprint in perpetuity. It is made available under aCC-BY 4.0 International license.

gRNA 5 (Supplemental Figure 6B)	CCTTCATAACTTGCCAACAA
gRNA 6 (Supplemental Figure 6B)	TTGCCACGTCTGGATGCTCA
gRNA 7 (Supplemental Figure 6B)	TTACCATGAGCATCCAGACG
gRNA 8 (Supplemental Figure 6B)	GTAAAAGAGATTAGTAACCC
gRNA 9 (Supplemental Figure 6B)	GGAGGAGGCACAGTCATCCT
gRNA 10 (Supplemental Figure 6B)	AGGGAAGTAGGCTATGCAGG
